# Gut–Lung Microbiota Interaction in COPD Patients: A Literature Review

**DOI:** 10.3390/medicina58121760

**Published:** 2022-11-30

**Authors:** Angelika Krumina, Marina Bogdanova, Sandra Gintere, Ludmila Viksna

**Affiliations:** 1 Department of Infectology, Riga Stradiņš University, 16 Dzirciema Street, LV-1007 Riga, Latvia; 2 Faculty of Residency, Riga Stradiņš University, 16 Dzirciema Street, LV-1007 Riga, Latvia; 3 Department of Family Medicine, Riga Stradiņš University, 16 Dzirciema Street, LV-1007 Riga, Latvia

**Keywords:** COPD, microbiota, chronic obstructive lung disease, dysbiosis, lung, immunity, bacteria

## Abstract

Respiratory diseases are one of the leading causes of death in the world, which is why a lot of attention has been recently paid to studying the possible mechanisms for the development of pulmonary diseases and assessing the impact on their course. The microbiota plays an important role in these processes and influences the functionality of the human immune system. Thus, alterations in the normal microflora contribute to a reduction in immunity and a more severe course of diseases. In this review, we summarized the information about gut and lung microbiota interactions with particular attention to their influence on the course of chronic obstructive pulmonary disease (COPD).

## 1. Introduction

The “microbiota” is a term for all microorganisms that live in or on the human body (bacteria, viruses, fungi, parasites, and archaea), and it contains both symbiotic and pathogenic microbes. The microbiome is the genetic and functional profile of these microorganisms [1].

The gastrointestinal tract is populated by the largest microbial community that consists of trillions of microorganisms, dominated by the *Bacteroidetes*, *Firmicutes*, *Actinobacteria*, *Proteobacteria*, and *Verrucomicrobia* [2].

**Figure 1:**
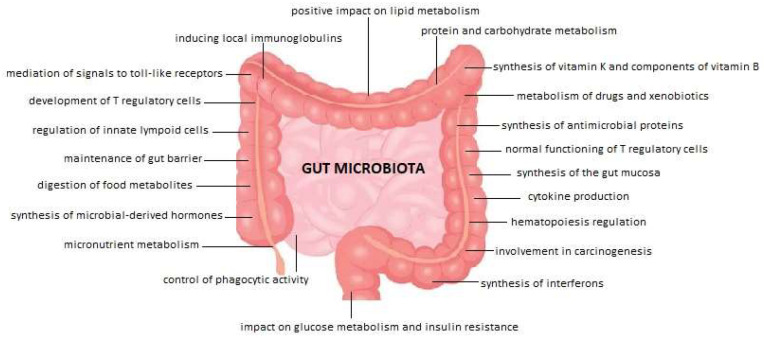
The main functions of the normal gut microbiota.

Gut microbiota plays an important role in many organs’ functions, for example, development and modulation of the immune system, digestion and intestinal metabolism, production of vitamins and regulation of inflammation [3]. The main functions of the microbiota are summarized in Figure 1 [4,5,6].

Intestinal microbiota has systemic effects on the human body interacting with other organs. The bidirectional interactions between the gut microbiota and respiratory mucosa are known as gut–lung axis [7]. In addition to microbes, their mucosal immunity and metabolites have also been studied in the gut–lung axis [8]. Changes in the gut microbial community and functions called dysbiosis could result in different intestinal diseases as well as secondarily affect lung function and severity of respiratory diseases through this relationship.

Statistics show that up to 33% of patients with irritable bowel syndrome and 50% of patients with inflammatory bowel disease have respiratory inflammation or function loss even without a medical history of acute or chronic lung diseases. Furthermore, the frequency of inflammatory bowel diseases in chronic obstructive pulmonary disease patients is 2–3 times more common than in the general population [9].

According to World Health Organization (WHO), respiratory diseases are one of the leading causes of death worldwide [10]. Scientists pay a lot of attention to the influence of the microbiota on the development of bronchial asthma [11,12], cystic fibrosis [13,14] or COVID-19 [15,16]. We decided to focus on chronic obstructive pulmonary disease, because it is prevalent in the adult population and is one of the leading causes of death worldwide [17]. Uncontrolled COPD causes disability in many patients and it is a major burden on the healthcare system. Therefore, we felt it was important to better understand its pathogenesis in the context of the gut–lung axis.

## 2. Characteristics of COPD

According to WHO data, chronic obstructive pulmonary disease is the third leading cause of death worldwide with 3.23 million deaths in 2019 [17]. Approximately 10% of the world’s population suffers from this disease [18]. The progressive pollution of the environment and the growing number of smokers give a disappointing prognosis for future perspectives. The European Respiratory Society has published that in 2050 there will be 49,453,852 people in Europe suffering from COPD (prevalence is 9.3%). This means that compared to 2020 the prevalence will increase by 39.6% [19].

COPD is characterized by persistent respiratory symptoms (dyspnea, cough with sputum, wheeze) with acute exacerbations and progressive, irreversible airflow obstruction. These processes are leading to the reduced lung functional capacity [20,21]. The inflammation in COPD is characterized by an increased count of neutrophil leukocytes, alveolar macrophages, and T lymphocytes. These cells secrete different proinflammatory mediators, which maintain chronic inflammatory reaction [22].

COPD is a multicomponent disease and has multifactorial etiology. Tobacco smoking is recognized as the general risk factor of COPD but different epidemiological studies report that about 30–65% of patients with COPD had never smoked. Other risk factors include passive smoking, air pollution, occupational exposure to dust, noxious gases and fumes, and genetic factors, for example, alpha-1 antitrypsin deficiency [21,23,24].

Many previously reported studies have demonstrated that patients with COPD more often have inflammatory bowel diseases. The risk of ulcerative colitis or Crohn’s disease development in patients with COPD is significantly increased compared to the healthy control [25,26,27]. Nowadays, there is increasing evidence that changes in the normal microbiome play an important role in the combination of these diseases because cigarette smoking not only affects the lungs but also causes changes in the microbiota of the distal gut [28,29].

It is known that COPD is related to decreased diversity of normal lung microbiota with replacement of resident flora by potentially pathogenic microorganisms [30]. In the healthy lungs, the predominant phyla are *Bacteroides* and *Firmicutes* [31]. In turn, COPD patients have an increased count of *Proteobacteria* [32,33]. In the stable state of COPD, the most common respiratory microorganisms are non-typeable *H. influenzae*, *M. catarrhalis*, and *S. pneumoniae* [34].

COPD exacerbations’ frequency is directly related to respiratory infections and the proliferation of pathogenic bacteria. Acute respiratory infections in COPD patients result in enlarged local and later also systemic inflammation, mucus hypersecretion with edema, bronchoconstriction, as well as decreased expiratory flow [35]. Even though viruses are the most common cause of exacerbation, bacterial infections also worsen the severity of symptoms [35,36,37]. Millares and co-workers analyzed sputum samples from COPD patients in a period of clinical stability and during exacerbations. They found increases in the relative abundance of *Haemophilus*, *Pseudomonas*, and *Moraxella* in exacerbations [38]. A heightened level of *Moraxella* spp. in the lung has been also demonstrated in other studies [39,40]. Another study done by Sun et al. shows that in exacerbated patients, there is a growth of the relative abundance of *Proteobacteria* and a reduction of *Firmicutes*. Disturbance in pulmonary microflora leads to the outgrowth of pathogens such as *Acinetobacter* and *Klebsiella* spp., that indicate the importance of the commensal microbiota in protection against colonization by pathogenic microorganisms [41]. Bacterial exacerbations are also characterized by a decrease in *Streptococcus* spp. [21].

The most widespread identified viruses during COPD exacerbations are *Human rhinovirus* and *Influenza virus* [35,39,42], but it is also reported about *Cytomegalovirus* and *Epstein-Barr virus* in the lung of COPD patients [21,43].

Moreover, an increase in the relative abundance of fungal pathogens is present in COPD patients, including *Candida* spp., *Aspergillus*, *Penicillinum*, *Cladosporium*, *Phialosimplex*, and *Eutypella* [39,44].

With knowledge about infections that can cause more severe forms of COPD, the vaccination against influenza and streptococcal infections is recommended for the prevention of COPD exacerbations [45].

**Figure 2:**
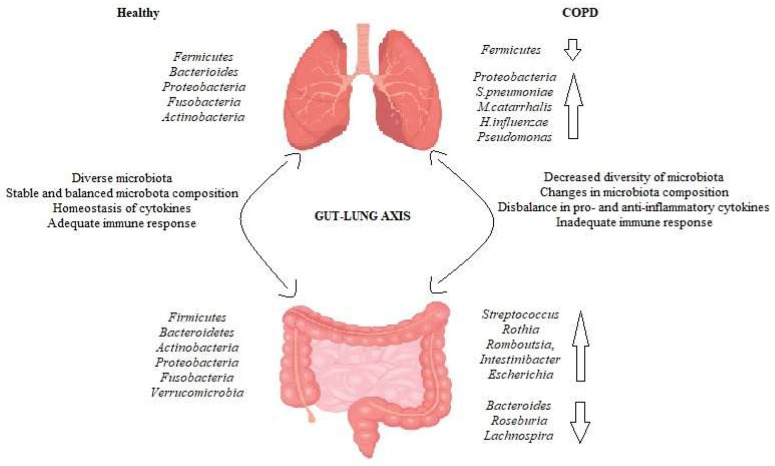
Changes in gut and lung microbiota during the COPD.

It is important to note that in COPD patients the microflora changes not only in the lungs but also in the intestinal tract. Bowerman and co-workers compared the gut microflora of COPD patients and healthy controls, and found that the abundance of *Streptococcus*, *Rothia*, *Romboutsia*, *Intestinibacter*, and *Escherichia* increased in COPD, but that of *Bacteroides*, *Roseburia*, and *Lachnospira* had reduced. Several species of *Streptococcus* and *Lachnospiracae* also correlated with reduced lung function [46]. In another study Chiu et al. investigated how the gut microbiota differs depending on the severity of COPD. The data showed that in severe COPD patients, the genera *Aerococcus* and *Fusobacterium* were the most abundant, while *Ruminococcaceae* and *Lachnoclostridium* were less abundant. However, the study did not find a correlation between gut microflora and COPD severity [47]. The most common changes in lung and gut microbiota during the COPD are demonstrated in Figure 2.

Although the exact mechanisms by which the microorganisms affect the course of COPD are still unknown, there is evidence that regulation of COPD severity by microbiota is associated with NLRP3 inflammasome which promotes inflammatory cell recruitment and regulates immune responses in the gastrointestinal and respiratory tracts as well as maintaining the epithelial barrier [48,49]. Commensal bacteria and its metabolite, called short-chain fatty acids (SCFAs), activate NLRP3 inflammasome which results in immune cells activation and inflammation in the lung to protect the human organism [48].

## 3. The Impact of COPD on the Intestinal Microflora

One of the main risk factors for developing COPD is smoking. Tobacco is known to have an immunosuppressive effect by decreasing the activity of natural killer cells, increasing the number of neutrophil and eosinophil leucocytes, and decreasing the phagocytic function of macrophages and neutrophils. These mechanisms prevent effective infection control, which can lead to the colonization of new bacteria and intestinal dysbiosis [50]. Lee and his co-authors demonstrated in their study that smokers have an increase of *Bacteroidetes* and a decrease in *Firmicutes* and *Proteobacteria* compared to non-smokers [51].

Long-term smoking can also reduce the oxygen level in the human organism, leading to the proliferation of anaerobic and microaerophilic bacteria. Shanahan et al. compared the duodenal microbiome in smokers and people who had never smoked. The study demonstrated that smokers had fewer *Prevotella* and *Neisseria* spp. and more *Firmicutes*, especially *Streptococcus* spp., and *Veillonella* spp. [52]. Rogers and colleagues examined in another study that smoking is associated with a higher risk of infection with *Clostridiodes difficile*, which is also an anaerobic bacterium [53].

In general, oxygen is one of the key factors in maintaining the homeostasis of the gut microbiota. The level of intestinal oxygen depends on two processes: oxygen supply from the bloodstream and oxygen consumption in the gut [54]. Microbiota-derived metabolites, e.g., SCFAs, which will be discussed in more detail below, increase the oxygen consumption of epithelial cells, leading to physiological hypoxia in the gut [55]. The response to hypoxia is mediated by hypoxia-inducible factors (HIFs), which regulates the adaptation to the reduced oxygen level [56]. However, systemic hypoxia in COPD patients exacerbates pre-existing hypoxia in the intestine, leading to disruption of adaptive mechanisms and dysbiosis. The effect of hypoxia on gut microflora has been described in many studies. For example, Adak et al. demonstrated that the total number of anaerobic bacteria increases, and the number of aerobic microorganisms decreases in the faecal microflora of people exposed to high altitudes. In addition to the composition of the microbiota, the enzymatic activity of the microbes also changed under the influence of hypoxia [57]. Van Meijel et al. recently investigated the changes in the intestinal microflora under the influence of mild intermittent hypoxia in overweight men. The results of the study also showed a higher abundance of anaerobic butyrate-producing bacteria [58].

It is also important to note that COPD patients mostly have a sedentary lifestyle, but physical activity also has an impact on the intestinal microbiota. It is known that physical activity is associated with a greater diversity of intestinal microflora [59], therefore sedentary people have a less diverse microflora. Jollet and co-workers conducted a study aimed at investigating the effects of hypoactivity on microflora. For this purpose, 14 men were subjected to five days of dry immersion, a model of severe hypoactivity. Bacteria of the *Fermicutes* phyla, namely *Clostridiales* and *Lachnospiraceae* were significantly increased after a short period of physical inactivity [60].

During exacerbations of COPD, many patients need to take antibiotics, which also cause gut dysbiosis. The use of antibiotics has been associated with reduced diversity of the microbiota [61]. Palleja et al. demonstrated that administration of meropenem, vancomycin, and gentamicin in adults results in an increased amount of *Enterobacteriaceae* and the depletion of *Bifidobacteria* and butyrate-producing bacteria. It should be said that most of the microflora recovered after 1.5 months, however, several species were still not detected at the end of the study (after 180 days) [62]. In another study, Kabbani and co-workers showed that the use of amoxicillin/clavulanic acid increased the prevalence of in *Escherichia*, *Parabacteroides*, and *Enterobacter* and reduced the amount of *Roseburia* [63]. On the other hand, the use of ceftriaxone and cefotaxime decreased the count of Gram-negative bacteria [64].

Antibiotic use is the most significant risk factor for the development of *C. difficile* infection. The most associated antibiotics are amoxicillin, ampicillin, cephalosporins, clindamycin, and fluoroquinolones [65].

Another mechanism of influence on the intestinal microflora is the destruction of microbes that are sensitive to antibiotics, and microorganisms that exhibit antibacterial resistance taking their place [61]. Antibiotic resistance is the capacity of bacteria to survive when antibiotics are used in a concentration that kill or inhibit other bacteria of the same species [66], which also leads to dysbiosis.

COPD patients must take glucocorticoids, which also interact with the gut microflora. Schepper and co-authors presented data showing that an 8-week course of prednisolone altered the abundance of *Bacteriodales* and *Verrucomicobiales* phyla in mice [67]. Another study demonstrated that the effect of prednisolone on the gut microbiota in mice was dose-dependent, but in general, corticosteroid use was characterized by an increase in *Parasutterella* and a decrease in *Rikenella*, *Christensenella*, *Ruminococcus*, and *Intestinimonas* [68].

## 4. The Role of the Gastrointestinal Tract in the Immunity

The gastrointestinal and respiratory systems’ diseases often overlap each other. It should be noted that both organ systems have many common features. Both lung and intestinal tissue embryonically developed from the primitive foregut [8]. Normally, gut and lung have a stable microbiome and a big epithelial surface area. Mucosal tissues act as the primary innate and adaptive immunity against foreign pathogens [2,69]. They both contain mucosa-associated lymphoid tissues that are significant for the immune defense [70,71]. This can explain the close interactions of these organ systems in ensuring the body’s homeostasis.

Gastrointestinal tract and gut microbiota play an important role in the normal functioning of the immune system. There are different anatomical and morphological structures in the intestine responsible for various functions to ensure the protection of the human organism. The intestinal epithelial layer is the major physical barrier that separates the human body from the external environment [72]. The epithelium consists of different specialized cells. Disturbances in the structures of the intestinal barrier can lead to an uncontrolled change in the microbiota which threatens the development of both intestinal and extraintestinal diseases including autoimmune pathologies [73,74]. The enterocytes (including Paneth, Goblet, Microfold cells) make up about 80% of the total epithelial cells. Their main functions are to absorb nutrients and maintain the integrity of the epithelial layer, which prevents the pathogens and toxins from entering the deeper layers of the intestine. Epithelial cells also produce different cytokines, chemokines, and antimicrobial peptides (AMPs) [72,75,76,77,78,79]. All these cells are connected by tight junction proteins including claudins, zonula occludens, tricellulin, occludin, cingulin, and junctional adhesion molecules which form a complex gut barrier to regulate microbiota–host relationships [72,78].

Incorrect diet, the recent use of antibiotics, the presence of infections or allergens and nicotine exposure can cause changes in normal gut microflora called dysbiosis [80,81]. There are diversified mechanisms that disrupt the normal protective function of the body in the case of intestinal dysbiosis.

## 5. The Mechanisms of Gut–Lung Interactions

One of the regulatory mechanisms is the control of the secretion and activity of cytokines. The gut microbiota is involved in the expression of type I interferon receptors (IFN α/β) in the epithelium of the gut and airways. Interferon is responsible for antiviral immunity [82]. It has been previously reported that the microbiota-induced signals to lung stroma provide homeostasis of interferon activity in the pulmonary tract and prevent the development of viral respiratory infections like influenza by early control of virus replication [83,84]. Further investigations are necessary to understand the nature of these signals, but it is already published that some metabolites of gut bacteria can affect the production and activity of IFN-1. Steed et al. demonstrated that *Clostridium orbiscindens* produces desaminotyrosine, which has a stimulatory effect on the IFN-1 protecting against the influenza infection [84]. Another study shows that commensal microbiota has both local and systemic effects on the INF-1 through secretion of INF-β by dendritic cells in the colon. It is identified that *Bacteroides fragilis* capsular polysaccharide A (PSA) through toll-like receptor 4 (TLR4) signaling can stimulate INF-β expression [85]. It should be noted that PSA not only affects the metabolism of interferon but also induces the production of anti-inflammatory Il-10 by T- lymphocytes [9].

The microorganisms and their components can get into the systemic circulation reaching the lungs through the intestinal lymphatic system. Increased intestinal permeability drives inflammatory cells migration to extraintestinal organs and occurrence of systemic inflammation processes. Lipopolysaccharides (LPS) (also known as endotoxins) is the cell wall component of Gram-negative bacteria, which can induce acute lung injury [86]. In healthy people, the gut barrier limits LPS migration to systemic circulation [87]. The pathology of the gut barrier allows LPSs to enter the bloodstream and start inflammatory reactions either by binding to the LPS-binding protein or by attaching to receptors on the surface of immune cells (TLR4 and MD-2). Processes following the formation of this receptor complex include intracellular signaling via MyD88 and TRIF with the consequent activation of nuclear factor-kB (NF-kB). It results in proinflammatory cytokines transcription such as IL-1β, IL-6, and TNFα. A further response to these pro-inflammatory processes is the activation of macrophages and the infiltration of immune cells, which enlarge inflammation [88,89,90]. LPS is one of the complement activators, which also contributes to inflammation [86].

An incorrect diet also effects gut microbiota that results in changed SCFAs profile. SCFAs are fatty acids’ molecules which are produced by dietary fibers (partially and nondigestible polysaccharides) during bacterial fermentation. The main sources of dietary fibers are grains, vegetables, fruits, seeds, and nuts. It is known that high intake of dietary fibers can reduce the incidence of COPD in smokers [91,92]. In another study, Jang et al. demonstrated that a diet with high level of dietary fibers had a beneficial effect on emphysema in cigarette smoke-exposed mice by reducing alveolar destruction and inflammation [93].

SCFAs are substantial for immune homeostasis in many ways: they save the integrity of the intestinal epithelium, increase production of IgA, stimulate Il-10 synthesis, and increase the number of Goblet cells, which produce mucin [78]. SCFAs are also affecting the amount and activity of Th1, Th17 and regulatory T cells (Treg) [94]. They penetrate the bone marrow and affect differentiation of dendritic cells and macrophages precursors [78]. Surveys show that magnification in precursor cells causes elevation of Ly6c- monocytes in the bone marrow, blood, and lungs. Ly6c- cells can differentiate into alternatively activated macrophages (AAMs) to repair tissue damage. SCFAs also improve CD8+ cell metabolism during influenza infection that results in more effective viral clearance [95].

An SCFA called butyrate, which is produced by *Faecalibacterium*, minimizes LPS translocation in the systemic circulation, reducing the LPS-associated effects described above. Butyrate also inhibits pro-inflammatory reactions by suppressing the activity of NF-kB, which is responsible for pro-inflammatory regulation [87,96,97].

Another way to inhibit inflammation is to promote FOXP3 expression—a transcription factor which represses the production of Il-9. Vieira and co-workers demonstrated that butyrate is effective in promoting FOXP3 expression [98]. Moreover, butyrate suppresses degranulation of the mast cells in the gut mucosa and limits circulating inflammatory mediators’ production [99]. The reduction of butyrate-producing bacteria also leads to a decreased level of Il-22, which is necessary for a normal gut and lung epithelial barrier. [100].

Another SCFA called propionate, which is produced by *Phascolarctobacterium*, has the same effect on NF-κB as butyrate [101,102].

Acetate is SCFA produced by *Bifidobacteria* which modulates inflammation by activating G protein-coupled receptor 3 (GPR43) [103]. During the research it was found that acetate protects mice from respiratory syncytial virus infection by elevated IFN-β producing in lung epithelium through GPR43 in neutrophils and alveolar macrophages [104,105]. The activation of GPR43 also takes a part in the control of pulmonary infection caused by *Klebsiella pneumoniae* [106] and *Streptococcus pneumoniae* serotype 1 in mice [107]. Acetate promotes anti-inflammatory reactions by diminishing NF-kB activation and suppressing pro-inflammatory mediators such as lipopolysaccharide-induced TNF-α [103,108].

Other gut microbiota metabolites are extracellular vesicles (EVs)—structures with proteins, glycolipids, polysaccharides, phospholipids, and nucleic acids. EVs take a part in immunomodulation as well as signaling and communication between cells. There are outer membrane vesicles (OMVs) which are produced by Gram-negative bacteria and membrane vesicles (MVs) produced by Gram-positive microbes [109]. It is known that OMVs from *Bacteroides fragilis* can minimize the immune response by affecting Toll-like receptor-2 and -4 and expanding anti-inflammatory cytokines levels [110]. There are already published data that *Akkermansia muciniphila*—derived EVs—can improve the intestinal barrier because they are rising tight junctions [111,112]. *Akkermansia muciniphila* EVs also affect Toll-like receptor-2 and -4 activity, expression of pro-inflammatory TNF-α and Il-8, and activation of anti-inflammatory Il-10 [112]. These mechanisms are important for maintaining immune homeostasis between pro and anti-inflammatory cytokines expression.

Another mechanism of influence on the immune system is the effects of various substances produced by bacteria. The majority of commensal bacteria which have enzyme tryptophanase can produce indole that improves the epithelial barrier in the colon [113]. Tryptophan’s metabolites can inhibit the immune system to protect organisms against expressive inflammation and induce long term immune tolerance [114]. Its metabolite tryptophol significantly lessens production of IFN-γ and inhibits TNF-α response [115].

It is interesting that new studies about COVID-19 pathogenesis have shown that angiotensin-converting enzyme 2 (ACE2) has an impact on the development of a severe form of the disease. There is information that a deficiency of ACE2 in mice leads to disruption of local tryptophan homeostasis which makes the animals more susceptible to inflammation [8,116]. Fanos and co-workers also published a hypothesis that probiotics may ease COVID-19 disease severity because of ACE2 localization in the intestinal epithelium. This way gut dysbiosis can influence ACE2 level [117].

One more way to influence the immune system is the regulation of hematopoiesis. Commensal microbiotas regulate myeloid homeostasis without a state of infection or inflammation. Regulation occurs through various signal transductions such as Nod1, which stimulates the release of Il-17 to delay apoptosis of neutrophils and mononuclear phagocytes [118]. In turn, Nod2 signal transmission promotes the recognition of pathogenic microbes in the context of inflammatory bowel diseases [34].

There is evidence that gut bacteria in the normal state can stimulate the production of symbiotic bacteria-specific IgG, which provides systemic protection in case of violation of the intestinal barrier function and entry of symbiotic bacteria into the systemic circulation. It is not completely clear, what mechanisms provide synthesis under homeostasis conditions, but Zeng et al. found that Gram-negative bacteria have murein lipoprotein in their membranes, which is one of the major antigens that induces homeostatically the systemic IgG. It is an essential protective factor in term of gram-negative systemic infections being effective against salmonellosis and *E. coli* [119].

Another example of stimulating systemic inflammation by gut flora is *Proteobacteria* spp. These microbes have a positive correlation with increased level of Il-6 and Il-8 [120]. Another possibility of influencing the immune system is the effect on T-cells. Th17-associated immunity in the gastrointestinal tract is dependent on the presence of segmented filamentous bacteria (SFB) in the gut microflora. SFB are the first identified commensal bacteria that impact the growth of Th17 cells in mice. Defects in Th17 signaling are associated with the high risk of staphylococcal infections in the lung and skin. [121].

Th17 cells are the main producers of Il-22 and Il-17A. Both regulate the production of CXC and granulocyte colony-stimulating factors in the lung but only IL-22 increases lung transepithelial resistance to injury. Gauguet at al. foundthat SFB-colonized mice have a milder pneumonia caused by Methicillin-resistant *Staphylococcus aureus* (MRSA) They proved that mice with a higher amount of SFB in the gut have also a higher level of Th17 effectors in the lung and increased Il-22, resulting in the protection against severe pulmonary staphylococcal infection [122].

It was previously reported that Th17 has a role in the mucosal defense against other pathogens such as *Klebsiella pneumoniae* [123], *Pseudomona aeruginosa*, *Mycobacterium tuberculosis*, *Mycoplasma pneumoniae*, and *Chlamydia pneumoniae* [124,125]. SFB also activates IgA production and the development of lymphoid tissues [126,127].

Nowadays, there is a rising number of people who suffer from *Helicobacter pylori* which lives in the stomach. This bacterium is associated with a great risk of gastroduodenal ulcers, stomach cancer, developing COPD, and with reduced lung function [128,129].

## 6. Probiotics—Possible Components of Therapy?

The gut microbiota has many ways of influencing the normal functioning of the immune system. Any change in the normal flora can lead to increased inflammatory reactions that may occur in the lungs as well. Therefore, high attention has recently been paid to the possibilities of probiotics to improve the course of various diseases.

Probiotics are live microorganisms that are intended to have health benefits when they are taken in optimal quantities. They usually can be found in yogurt and other fermented food and dietary supplements. Lactic acid bacteria (*Bifidobacterium* and *Lactobacterium genera*) have many positive effects through the suppression of the opportunistic gut bacteria. Probiotics can also be a part in immunomodulation processes, signal transduction and the saving of the epithelial barrier [130,131,132].

Probiotics have a positive effect on influenza virus infection. Jung et al. published data showing that heat-killed *Lactobacillus casei DK128* decreases influenza viral load in mice [133]. Another probiotic from the *Lactobacillus* family—*L. paracasei CNCM I-1518* strain—also showed efficiency against the influenza virus in a study with mice. Belkacem and co-authors found that this probiotic modulates lung immunity and that mice were less susceptible to the influenza infection after peroral receiving of *L. paracasei* [134]. Moreover, probiotics *Lactobacillus paracasei* and *casei 431* and *Lactobacillus fermentum PCC* may diminish the duration and symptoms of flu and other respiratory tract infections by increasing the level of IFN-g in the blood and IgA in the intestinal tract [135].

There is also data that *Lactobacillus casei strain Shirota* oral intake activates the natural killers. It is especially important for patients with COPD who are smokers because smoking has a negative effect on natural killer cells’ activity [136]. Reale at al. also proved a positive effect of *L. casei Shirota* on NK cytotoxic activity in male smokers’ population [137].

Salva et al. demonstrated *Lactobacillus rhamnosus’* resistant effect against *Streptococcus pneumoniae* infection by reducing bacterial load in the lungs and increasing INFγ, IL-6, IL-4, and IL-10 in bronchoalveolar lavage [138]. A symbiotic composed of *Lactobacillus rhamnosus GG ATCC5310*, galactooligosaccharides, and polydextrose, also reduced Rhinovirus infection rate in preterm infants as it was shown in the randomized, double-blind, placebo-controlled study [139].

Talking about another member of the lactobacillus family called *L. plantarum*, it is worth noting the article written by Anwar et al. about preventing the possible antiviral effects of this probiotic’s metabolites against SARS-CoV-2 infection by preventing the virus from binding with ACE2 receptors [140]. One more study demonstrated that the oral administration of the yogurt supplemented with *Lactobacillus paracasei N1115* can reduce the risk of upper respiratory tract infection in the mid-aged and elderly patients by stimulation of the T cells [141]. It was concluded in the clinical trial that the use of probiotic bacteria *Bacillus subtilis* and *Enterococcus faecalis* significantly decreased the development of ventilator-associated pneumonia in critically ill patients [142]. But Shimizu et al. presented data about administration of a symbiotic consisting of *Bifidobacterium breve Yakult*, *L. casei Shirota* and galactooligosaccharides can lessen the incidence of ventilator-associated pneumonia in patients with sepsis in the intensive care unit [143].

COPD development and progression often are associated with the high expression of inflammatory mediators induced by cigarette smoke. One of them is CXCL-8 which is an important mediator of neutrophils’ recruitment and activation. CXCL-8 is controlled by a transcription factor called nuclear factor-kappa of activated B cells (NF-κB). The role of this factor in the processes of pulmonary immunomodulation caused by intestinal microflora has already been briefly described earlier. Mortaz et al. investigated changes in the expression of CXCL-8 from macrophages under the influence of probiotics such as *L. rhamnosus* and *Bifidobacterium breve*. Both inhibit NF-κB activity, which is activated by cigarette smoke resulting in suppressed inflammatory mediator release [144]. The dietary supplementation of *L. rhamnosus* and *B. breve* in COPD mice prevented the airway inflammation and damage by restoring the balance between cytokines and chemokines [145]. Furthermore, the use of live *Bifidobacteria longum 51A* in mice with *Klebsiella pneumonia* infection activates the TLR signaling pathway, which leads to the faster production of interleukin 10 and reduces bacterial load and lung damage [146].

Cigarette smoking can directly change the virulence and structure of microorganisms. For example, it affects exopolysaccharide of *Bifidobacterium animalis* which leads to dysbiosis. It is important that changes caused by smoke persist for a long time after smoking has been stopped, therefore, repeated administration of probiotics may be necessary to avoid dysbiosis even if the patient has stopped smoking [9].

## 7. Discussion

In recent years, there has appeared a huge interest in the study of immunity, namely the factors of the human body that affect the severity of certain diseases. There are well-known factors affecting immunity such as age, the presence of immunosuppressive conditions (HIV, oncology, diabetes mellitus, glucocorticoid therapy, and others), genetics, recent infectious diseases, and smoking [147]. However, the human microbiota and its impact on homeostasis, with a particular focus on gut flora, has become one of the popular areas of research. In addition to local reactions, the intestinal microflora has systemic effects on the development of diseases by interactions with other organs. The influence of the microbiota dysbiosis on the development of asthma, COPD, neurodegenerative diseases, diabetes mellitus, obesity, psoriasis, and others has been proven [148,149]. The study of the normal microbiome and the mechanisms of its influence on immunity can open great prospects for the prevention and treatment of diseases in the future.

There is evidence of the impact of diet [150,151] and exercise [152,153] on the microflora as well as the use of certain prebiotics and probiotics. Despite the positive effect of the addition of dietary supplements to the diet, there is not enough clinical evidence to include probiotics in standard treatment regimens. The World Gastroenterology Organization issued practice guidelines for the use of probiotics and prebiotics in 2017, but their use can be only justified in patients with diarrhea of various etiology so far [154].

However, published data indicate that a normal intestinal microbiota is needed not only for intestinal homeostasis, but also for the whole organism. Dysbiosis affects many processes that occur in the formation of an adequate immune response, such as cytokine response, cell differentiation in the bone marrow, activation of toll-like receptors, and immune cell activity.

Some works demonstrate the positive effect of specific probiotics on the course of respiratory diseases, but there are not enough systematic reviews and clinical studies so far to apply these theoretical data in practical work.

Indeed, it is known that smoking, which is a major risk factor for COPD, alters the intestinal and lung microbiota, thus reducing immunity. But there is very little data on the restoration of immune function by restoring the microflora through the use of probiotics. Although it is worth to point out that there are positive results of studies in the population of smokers. However, it is important to understand that the use of probiotics cannot be effective without smoking cessation because the effects of smoking in the body persist for a long time even after quitting tobacco.

Further research with a focus on COPD is needed to assess the possible prospects for the treatment and prevention of this disease by maintaining a normal intestinal microbiota.

## 8. Conclusions

Recently published data show that the homeostasis of the gut microbiota plays an important role in the maintenance of immunity. Changes in the microflora have not only a local effect on the gastrointestinal tract, but also systemic effects through bilateral interaction with other organs. This work demonstrates that gut dysbiosis may influence the course of COPD by affecting the synthesis and activity of various pro- and anti-inflammatory cytokines and immune cells, regulating hematopoiesis, or changing intestinal permeability. Studying the effects of the gut microbiota on human organism may be a promising avenue for new therapeutic possibilities.

Considering the growing number of patients with pulmonary diseases, this direction should be further explored.
